# Promoting Physical Activity and Nutrition Through Health Education: Insights from a Study on Middle Eastern Adolescents in Malaysia

**DOI:** 10.21315/mjms-10-2024-785

**Published:** 2025-06-30

**Authors:** Hanan Al-Haroni, Nik Daliana Nik Farid, Mohamad Shafiq Azanan

**Affiliations:** 1 Department of Social and Preventive Medicine, Faculty of Medicine, Universiti Malaya, Kuala Lumpur, Malaysia; 2 Department of Medicine, Faculty of Medicine and Health Sciences, Sana’a University, Sana’a, Yemen; 3 Centre for Population Health, Department of Social and Preventive Medicine, Faculty of Medicine, Universiti Malaya, Kuala Lumpur, Malaysia; 4 Department of Paediatrics, Faculty of Medicine, Universiti Malaya, Kuala Lumpur, Malaysia

**Keywords:** nutrition, physical activity, knowledge, attitude, practice, school students

## Abstract

**Background:**

This study aims to determine the effects of an intervention programme on knowledge, attitude, and practice of nutrition and physical activity, and physical activity level among Middle Eastern adolescent students in Arabic schools in Malaysia.

**Methods:**

A cluster randomised controlled trial was conducted among early adolescence, aged 13 and 14 years from February 2022 to September 2023. The study involved 250 participants, with 125 in the intervention group (IG) and 125 in the control group (CG). The IG underwent a “Healthy Lifestyle” educational programme, while the CG was not exposed to the programme. Knowledge, attitude, practice and physical activity level were assessed using questionnaires.

**Results:**

The study began with 250 participants divided into two groups. Dropouts occurred, resulting in 116 IG and 119 CG participants pre-intervention, 113 IG and 115 CG post-intervention, and 111 IG and 112 CG at follow-up. A significant intervention effect was found in improved knowledge, attitude, practice and physical activity level of the IG post-intervention and at the three-month follow-up compared to pre-intervention (*P* < 0.001). Specifically, the mean scores for knowledge, attitude, and practice of IG have significantly changed from the pre-intervention to follow-up, with mean differences of −13.03, −15.62, and −10.90, respectively, while physical activity levels changed from the pre-intervention to follow-up, with mean differences of −0.33 in the IG.

**Conclusion:**

The educational programme could increase awareness of nutrition, physical activity, and healthy behaviour among Middle Eastern adolescents, thereby potentially reducing obesity and non-communicable diseases among school students in the future.

## Introduction

Obesity is one of the biggest public health problems of the twenty-first century. Overweight and obesity are serious problems not only in high-income countries but also, increasingly, in low- and middle-income countries, especially in urban areas. More than 1.3 billion people are obese, and this number is expected to rise to 2 billion by 2030 (1–3). Childhood overweight and obesity have increased in recent decades: in 2016, an estimated 124 million children and adolescents aged 5 to 19 years suffered from obesity, and 213 million were overweight (4). According to the Centres for Disease Control and Prevention (CDC), obesity prevalence among adolescents aged 12 to 19 years increased from 5% in 1980 to over 21% in 2012 (5). More recent data from 2022 to 2023 indicate that about 17% of youths aged 6 to 17 years are obese (6). This trend highlights the ongoing challenge of adolescent obesity.

A study among Yemeni adolescents aged 12 to 18 years living in Selangor and Putrajaya, Malaysia, found that the prevalence of overweight and obesity was 23.6%, highlighting the elevated level, which is notably higher than the prevalence reported among adolescents in Yemen itself. This suggests that the transition to the Malaysian environment, with greater access to high-calorie foods and more sedentary lifestyles, may contribute to this population’s increased weight issues (7).

Adolescents undergo various biological, psychological and cognitive changes that can affect their eating habits and nutritional needs. Nowadays, eating out, snacking and skipping meals are common eating habits among adolescents, which are exacerbated by a nutrient-poor diet (8). The eating habits of adolescents have changed worldwide due to urbanisation and globalisation, resulting in a shift from traditional foods to fast food and convenience foods and a decrease in fruit and vegetable servings (9). Numerous factors, such as lack of exercise, inactivity and socioeconomic status, are associated with obesity and overweight (2, 9). Obesity, in general, is associated with several non-communicable diseases (NCDs) and premature mortality (2). According to the WHO, several countries are at risk of NCDs due to the growing obesity epidemic (3).

Moreover, physical activity levels among Middle Eastern adolescents are a significant concern due to the high prevalence of physical inactivity in the region. Studies indicate that a substantial portion of adolescents in the Middle East do not meet recommended physical activity levels, which can lead to health issues such as obesity and other NCDs (10, 11). Obesity in childhood and adolescence is associated with a variety of short- and long-term health problems. Over time, obesity can lead to adverse effects such as increased blood lipids, altered blood glucose metabolism and obstructive sleep apnoea, as well as more serious consequences such as diabetes, hypertension, gallbladder disease and osteoarthritis (12).

A systematic review of childhood obesity in the Middle East and North Africa (MENA) region found that there is a significant association between childhood obesity and negative health outcomes such as higher blood pressure, prediabetes, metabolic abnormalities, cardiovascular risk, dental decay, mental health problems and lower health-related quality of life. The results also showed that the interventions achieved variable results and had little to no overall impact on childhood obesity rates. In addition, the study included various management and intervention strategies, including school-based programmes, clinical services, educational resources and lifestyle changes, as well as the effectiveness of these interventions. The findings indicated promising outcomes of some interventions, highlighting the need for multifaceted approaches targeting individual, family, and policy levels (13).

Although school-based physical activity programmes are one of the best sources for promoting physical activity and fitness, adolescents are not adequately active (14). A variety of environmental and sociocultural factors, including family characteristics, parental lifestyle, school policies and screen culture, could influence eating and physical activity behaviour (14). As a result, designing and developing comprehensive school-based programmes to improve physical activity and nutrition behaviour among Middle Eastern adolescents in Malaysia is crucial for addressing the rising concerns of obesity and related health issues.

However, there have been no studies on educational interventions among Middle Eastern adolescents in Malaysia. As young adolescents, those aged 12 to 14 are at the best age to understand healthy eating and physical activity (15, 16). This study included 13 to 14 years old students attending intermediate school. The current study aims to determine the impact of a health educational intervention programme on nutrition and physical activity knowledge, attitudes and practices as well as physical activity levels among Middle Eastern students in Arab schools in Malaysia.

## Methods

### Study Design, Sample and Population

A cluster randomised controlled trial was conducted from February 2022 to September 2023 among Arabic students in early adolescence, at the age of 13 and 14 years (grades seven and eight, respectively), in four intermediate Arabic schools located in Klang Valley, Malaysia. The Klang Valley has 25 Arabic and International schools; four schools were selected and allocated from the 25 schools. The selection and allocation were done randomly through Excel software. Non-Arabic students, students with diseases on treatment such as asthma, diabetes, cancer, cardiovascular diseases, fractures, cirrhosis, or other diseases, and with physical disabilities or injuries that restrict physical activity were excluded from the study.

The formula for the Cluster Randomised Control Trial was used to calculate the sample size, taking into consideration a power of 80% and a 95% confidence level (17). According to the previous proportion of overweight among adolescents in a previous study (18), by adding 15 % to the number of subjects as an attrition rate, the total sample size is 250 participants (125 participants for each group).

There are 25 Arabic schools in the Klang Valley. Out of the 25 schools, four schools were selected and allocated according to the inclusion criteria. The inclusion criteria for selecting schools into the intervention and control groups were that the schools have certain resources, such as technology or specialised facilities to support the intervention, have more than 25 students in each classroom to ensure a representative sample, and that the schools agree to participate in the study and comply with the conditions for the intervention or control. These criteria ensure that the selected schools are comparable and suitable for the aims of the study.

The first two schools were assigned to the intervention school and labelled as the intervention group (IG), and the second two schools were assigned to the control school with the control group (CG). Four classes were randomly selected from each school (two classes consisted of seventh grade students who were 13 years old, and the second two classes consisted of eighth grade students who were mainly 14 years old). The selection and allocation of both schools and classes was randomised using sealed opaque envelopes by independent researcher.

A simple random sampling technique was used to select the 63 students aged 13 to 14 years from each school. The students were randomly selected using Excel software. For this selection process, the IDs of all students were entered into the datasheet, a random column was created, and the formula “= RAND()” was applied. Participants were assigned a unique code for the allocation of a masking mechanism. This measure served to avoid bias at the individual level.

### Educational Intervention

A comprehensive and multi-component educational obesity prevention programme called “Healthy Lifestyle” was developed based on previous studies (19, 20). The general objective of this intervention is to increase the knowledge, attitude and practice on healthy diet and physical activity among students by delivering education and behavioural skills. Participants were explained about the study’s objectives and the eligibility requirements for participation. The education programme was developed by the researcher after consultation with experts in health promotion with a particular interest in changing behaviour related to obesity.

The educational program was designed based on the health belief model (HBM) components to improve knowledge and promote healthy lifestyle adherence in terms of healthy diet behaviour and physical activity by focusing on the attitude and beliefs of students with increasing knowledge and perceived risk of diseases. This healthy lifestyle programme included educational booklets and educational classes. The educational booklet was distributed to them in the first session, and they were asked to take it home. They had to read it within one week. In the booklet, there were three chapters, including risk factors for NCDs, the need for a healthy diet and physical activity to reduce overweight and obesity, and how to make better decisions, boost self-esteem, and replace bad habits.

CG was not exposed to the programme, where they had their regular school curriculum and exercise routine. After the IG had completed the last session, the CG received all the materials from the Healthy Lifestyle education programme.

The programme for educational intervention was first created in English and translated into Arabic. The educational programme was designed based on the HBM, considering the recommendations from the Ministry of Health of Saudi Arabia (21). The educational programme’s content validity was established through a rigorous process involving six subject-matter experts from diverse fields, including public health, nursing and health behaviour specialists. The Item-Content Validity Index (I-CVI) threshold was set at ≥ 0.78, ensuring each item met the minimum agreement level (ratings of 3 or 4 on a 4-point relevance scale).

The Scale-Content Validity Index (S-CVI) required a ≥ 0.90 threshold, calculated as the average of all I-CVIs, to confirm overall coherence with programme objectives. After revisions based on expert feedback (e.g., simplifying language, removing irrelevant modules), the final I-CVI scores ranged from 0.80–1.00, and the S-CVI reached 0.94, exceeding recommended benchmarks. Experts also validated the Arabic translation for cultural and linguistic appropriateness, ensuring alignment with the original English version’s intent.

### Data Collection and Measures

Information was collected as a direct interview (face-to-face), and the research instruments data was divided into three parts: i) sociodemographic characteristics; ii) Knowledge, Attitude and Practice of Nutrition and Physical Activity Questionnaire (KAP-Q); and iii) Physical Activity Questionnaire for Older Children (PAQ-C). The questionnaires were originally developed in English and then translated from English into Arabic and then back-translated to English by two certified translators. The sociodemographic factors included information on participants’ age, gender, family size, household income, and parents’ education and occupations.

The KAP-Q was used to measure the knowledge, attitude, and behaviour of lifestyle related to diet and physical activity according to previous studies (19, 22). The KAP-Q consists of 73 items: knowledge (30 items), attitude (22 items), and practice (21 items). The presented knowledge part consists of 30 items to determine participants’ knowledge level on nutrition and physical activity. There are multiple-choice questions, and each choice has “True,” “False,” and “I do not know” response options. The answers were coded “1” for correct answers and “0” for incorrect answers, and “I do not know” response. There is a maximum score of 30 (all the multiple choices counted) and a minimum of 0. In the attitude section, there are 22 items with five Likert scales for assessing the attitude. There are five choices: strongly agree, agree, neutral, disagree, and strongly disagree. Eleven negatively termed questions were inversely recoded during data analysis. There is a maximum score of 110 and a minimum score of 22. A more positive attitude has higher scores.

Practice includes 21 questions with five Likert scales for assessing the practice. There are five choices, namely: very frequently, often, sometimes, rarely, and never. Nine negatively termed questions were inversely recoded during data analysis. There is a maximum score of 105 and a minimum of 21. A higher score shows more positive practice. The reliability of the Arabic version KAP-Q ranged between 0.888 and 0.977, based on Cronbach’s α (23).

PAQ-C was used in this study to calculate the physical activity levels of the adolescents. PAQ-C was selected to give an overview of physical activity levels among participants of this study because PAQ-C is suitable for school children (8 to 14 years old). The physical activity questionnaire for older children was developed and validated by Kowalski et al. (24). The PAQ-C is a self-supervised, seven-day questionnaire that calculates moderate to healthy physical activity levels during the school year (24, 25). The questionnaire was administered personally in a classroom during regular visits. PAQ-C has ten items. Each of the nine PAQ-C questionnaire items is scored from 1 (low physical activity) to 5 (high physical activity), and a mean score of all items constitutes the overall PAQ-C score.

As an illustration, item number 1 (rating 1 for “no” and 5 for “seven times or more”) on the activity checklist forms a composite score for item 1. Items from 2 to 8 (score 1 for the lowest and the highest activity response being 5) indicate that the answers for each question start from the lowest to the highest activity response. Item 9 needs to take the mean of all days of the week to form a composite score (score “none” being a 1, “very often” being a 5). Item 10 (PAQ-C) was not used as a part of the final active score; it instructs students if they were ill last week or if anything precluded them from doing regular physical work. Scores 1 to 5 for each of the nine items (items 1 to 9) can be used in the physical activity composite score by taking the mean of these nine items, which results in the final PAQ-C activity summary score (24). PAQ-C was shown to have adequate reliability based on Cronbach’s alpha of 0.777 (26). The mean of the total score between 1 and 2.33 indicates low physical activity, 2.34 to 3.66 shows moderate physical activity, and 3.67 to 5 indicates high physical activity (27).

All the participants were required to complete the self-administered questionnaire by following the instructions before the start of the programme (pre-intervention evaluation), after the final intervention session (post-intervention evaluation), and three months after intervention (three-month follow-up after intervention).

### Statistical Analysis

Data was analysed using Statistical Package for Social Sciences (SPSS) version 26 software for Microsoft Windows (Chicago, IL, USA). Pairwise comparison with Bonferroni correction was applied to compare the effect of groups and time points on knowledge, attitude, practices and physical activity. The effect sizes were categorised as “small, *d* = 0.2,” “medium, *d* = 0.5,” and “large, *d* = 0.8.” A Generalised Estimating Equation (GEE) was used to test the effect of the intervention programme on the selected variables (outcomes) between and within the group at pre-intervention, post-intervention, and three months after the intervention, which is adjusted for clustering effect. Missing data was not replaced. Outcomes were assessed at each time point along with a derived average overall of three-time points; thus, both the cumulative and overall effects are provided. A *P*-value less than 0.05 was deemed statistically significant.

### Ethical Considerations

Ethical approval for the randomised controlled trial was obtained from the Ethics Committee for Human Study of Universiti Malaya (UM.TNC2/UMREC_2039), and the directors of the selected schools. The trial was registered in the Clinical Trials Registration: www.ClinicalTrials.gov, identifier: NCT05694143.

Written consent was obtained from all parents of the participants and the participants before starting the study. Also, the participants and their parents were privy to a study description through an information sheet. The information provided covers the purpose and procedures of the study, the unrestricted right of a participant to withdraw from the study, and the data collection process. The participants and their parents were allowed to inquire about the research before extending their consent. Participants were assured that their participation in the study was entirely voluntary and that they had the unrestricted right to withdraw from the study at any time. All participants’ information was kept confidential by coding the questionnaires.

## Results

**Figure 1:**
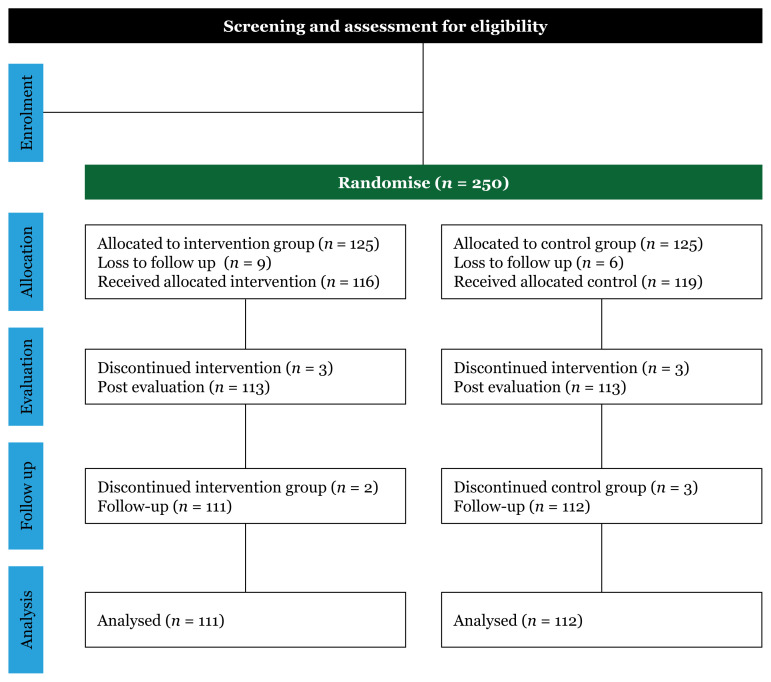
CONSORT flow diagram of the study

Figure 1 shows the CONSORT flowchart of the study. A total of 250 participants were selected and randomly assigned (125 participants for each group). However, nine participants dropped out of the IG, and six dropped out of the CG because they were unable to complete the study or were not interested. Thus, there were 116 participants in the IG and 119 in the CG. During the post-intervention data collection, an additional three participants dropped out from the IG and four from the CG. Dropout reason was due to their inability to complete the study and failure to attend three or more sessions. At the three-month follow-up stage, 223 out of the 250 participants—that is, 95.7% (*n* = 111) of the IG and 94.1% (*n* = 112) of the CG—participated in this study.

The sociodemographic characteristics of the participants were recorded in Table 1. The median age (IQR) for the participants was 14 years old. A majority of participants were female (53.4%) in the IG and male (52.1%) in the CG. Most of the students were Saudis (57.8%) in IG and Yemeni (67.2%) in CG. In addition, the majority of parents in both groups had undergraduate degrees or higher. According to Table 1, significant differences were observed between the groups for categories, including (number of siblings, nationality and household income). Therefore, these variables were considered as covariates for all analyses to remove their probable effect on the research variables. However, there was no significant difference in other sociodemographic variables between both groups (age, number of households, mother’s education, and father’s education).

In addition, there were no significant differences between both groups in knowledge, attitude, practice and physical activity at the pre-intervention stage. Nevertheless, the pre-intervention scores were used as covariates for those statistically significant variables at pre-intervention. Table 2 shows the descriptive statistics (mean and standard deviation [SD]) of knowledge, attitude, practice and physical activity for both groups at all three recruitment time points.

To assess the effectiveness of the educational programme, GEE analysis was applied thrice to assess whether there were differences in the groups and recruitment time points in the knowledge, attitude, practice and physical activity of the participants. Table 3 shows the results of the GEE analysis of knowledge, attitude, practice and physical activity. The results of the GEE on the total score for knowledge among the participants showed that time had a significant effect (*χ*^2^ = 433.834, *P* < 0.001). The effect of the group (IG/CG) was also significant (*χ*^2^ = 199.259, *P* < 0.001). Additionally, the interaction between time and group was significant (*χ*^2^ = 225.302, *P* < 0.001), which indicates that the two groups had a different pattern at all three-time points (pre-intervention, post-intervention, and follow-up) for knowledge.

Similarly, for the attitude domain, time points (*χ*^2^ = 320.420, *P* < 0.001) and groups (*χ*^2^ = 142.382, *P* < 0.001) were both shown to exhibit significant effects. The interaction between the time points and groups was also significant (*χ*^2^ = 240.788, *P* < 0.001) for the attitude domain. The same outcomes were observed as time points, and groups were shown to have significant effects on practice score (*χ*^2^ = 266.964, *P* < 0.001 and *χ*^2^ = 51.142, *P* < 0.001 respectively) and physical activity domains (*χ*^2^ = 94.633, *P* < 0.001 and *χ*^2^ = 19.415, *P* < 0.001 respectively). The interaction between time points and groups was also statistically significant for both practice and physical activity domains (*χ*^2^ = 86.566, *P* < 0.001 and *χ*^2^ = 55.491, *P* < 0.001, respectively). Therefore, based on the GEE analysis, it can be inferred that time points and groups had a significant effect on knowledge, attitude, practice and physical activity domain.

A pairwise comparison with Bonferroni correction was applied to evaluate the differences in the levels of knowledge, attitude, practice and physical activity among the participants across time for both groups (Table 4). The differences in the scores for knowledge between time points in IG and CG were statistically different (*P* < 0.001), except for scores between post-intervention and follow-up for the IG group. The effect size (*d*) indicated that a large effect of time on knowledge was observed in both groups, with a greater magnitude of the effect size in the IG (*d* = 2.75) than in CG (*d* = 0.79). For the attitude score, we observed a significant difference between pre-intervention with post-intervention and pre-intervention with follow-up in IG (*P* < 0.001), but not for the other time points.

Overall, the effect size indicated that time point had a large effect on attitude in IG (*d* = 2.62) and a small effect in CG (*d* = 0.23). The trend remains the same for the practice and physical activity domain, whereby a greater effect size of time point was found in the IG (*d* = 1.94; *d* = 0.23 respectively) when compared to the CG (*d* = 0.57; *d* = 0.04 respectively). Therefore, these findings suggested that the Healthy Lifestyle educational programme successfully improved the participants’ score for knowledge, attitude and practice on healthy lifestyle and level of physical activity of the IG from pre-intervention to follow-up.

To compare the levels of knowledge, attitude, practice and physical activity between the two groups across the three periods, a post hoc test (Bonferroni) was applied (Table 5). The results revealed that the level of knowledge between the IG and CG during the pre-intervention was not statistically significant (*P* = 1.000), while the differences in post-intervention and follow-up were significant (*P* < 0.001) between the IG and CG. These results also showed that the level of attitude between both groups at pre-intervention was not statistically significant (*P* = 1.000). In contrast, the differences between the groups were significant in the post-intervention and three-month follow-up (*P* < 0.001). Moreover, the level of practice between both groups at pre-intervention was not statistically significant (*P* = 1.000), whereas the differences between the IG and CG at the post-intervention and three-month follow-up were significant (*P* < 0.001) among the participants.

The differences between the level of physical activity of the IG and the CG at pre-intervention were not statistically significant (*P* = 1.000). However, the differences post-intervention (*P* < 0.001) and at follow-up (*P* < 0.001) were statistically significant. The effect size for knowledge, attitude, practice and physical activity between the IG and CG was calculated at three different times. The results indicated that knowledge had a large effect at the post-intervention (*d* = 2.56) and at follow-up (*d* = 2.40), while it had a small effect (*d* = 0.22) at the pre-intervention stage. The results for attitude indicated that it had a large effect in the post-intervention (*d* = 1.88) and follow-up stage (*d* = 1.75), while it had a small effect (*d* = 0.21) at the pre-intervention stage.

The effect size results showed that practice had a small effect between the groups at pre-intervention (*d* = 0.14) and a large effect at the post-intervention (*d* = 1.41) and follow-up test (*d* = 1.00). The physical activity had a small effect size at pre-intervention (*d* = 0.13), while the effect size post-intervention (*d* = 0.78) and at follow-up test (*d* = 0.79) was larger than that at pre-intervention. Therefore, the overall changes of knowledge, attitude and practice scores, along with physical activity level, were improved in IG compared to CG at post-intervention and follow-up stages, indicating the potential effect of the Healthy Lifestyle intervention programme.

## Discussion

We hypothesised that following completion of the educational programme intervention, Middle Eastern adolescent students in Arabic schools in Malaysia would have an improved knowledge of, attitudes towards and practices regarding nutrition and physical activity level. It is believed that the HBM-based educational programme positively influenced the responses of adolescents and that it can be used as a tool to evaluate the effectiveness of the educational programme intervention on nutrition and physical activity level knowledge, attitudes and practices.

The current study reported that the educational programme intervention significantly increased the IG’s knowledge of, attitudes towards and practices regarding nutrition and physical activity, and the mean (SD) knowledge, attitudes, and practices of the IG increased from 11.37 (6.56) to 24.41 (4.68), 69.15 (6.54) to 84.77 (8.23) and 59.98 (9.58) to 70.88 (6.51), respectively, from the pre-intervention to the follow-up. Thus, implementing school instructional programmes could enhance students’ comprehension of and attitudes towards nutrition and physical exercise. The current study’s findings are consistent with findings from several other studies that have been carried out among adolescents in different countries (19, 28–31). It was found that educational programmes aimed at increasing knowledge of, attitudes towards and practices regarding nutrition and physical activity level had a positive impact on adolescents’ knowledge levels and attitudes. Therefore, the findings of this study are consistent with those of previous studies.

According to Alamri (29), the IG’s knowledge of, attitudes towards and practices regarding nutrition and physical activity level considerably outperformed those of the CG following the educational programme at the two-month follow-up (*P* = 0.05). In another study by Ali et al. (28), adolescents in the IG improved significantly in terms of their attitudes towards physical activity and diet compared to the CG after three months, and they improved their physical activity and diet practices and showed significant progress (*P* = 0.001). In addition, Florence et al. (32) reported that IG had significantly more positive attitudes towards diet than CG after an educational intervention (*P* = 0.001). A study by Darabi et al. (33) found that IG had more positive behaviour towards physical activity than CG (*P* = 0.001). Thus, educational programmes lasting longer than six months are sufficient to change teenagers’ knowledge, attitudes and practices.

Due to the relationship between knowledge and attitude, it is not surprising that the attitude of adolescents who participated in several ongoing studies improved after the educational programme (34). This could account for the favourable attitude shifts observed in the current study following the intervention. Despite these findings, Sharif Ishak et al. (22) and Fetohy et al. (31) found no discernible change in Arab teenagers’ nutrition awareness, attitudes, or practices regarding diet and physical activity. The differences between the current study and other Arab studies can be attributed to the fact that they were conducted in different communities with varying cultures, socioeconomic statuses, sampling techniques, population characteristics, and follow-up periods, as well as inconsistencies in study design and methodology.

In addition, the level of physical activity of the IG was positively influenced by the intervention educational programme, where physical activity scores of IG increased from 1.90 (0.59) to 2.23 (0.42) from pre-intervention to follow-up. The proportion of IG participants with a high level of physical activity increased significantly. The present findings were consistent with a study by Hefni (35), which investigated whether educational programmes can increase the physical activity levels of young Arab adolescents. Several other studies have found that educational programmes help children, adolescents, and their parents to adopt, change and maintain their physical activity behaviour (32, 36, 37). Teenagers can, therefore, be kept physically active by educating them about the dangers of inactivity and the benefits of physical activity and by applying the skills learned in the educational programme and engaging in physical activity (36). There is evidence that using the HBM to create physical activity behaviours can improve physical activity behaviours, as demonstrated by numerous educational programmes that have used it (22, 36, 37).

The health educational intervention findings in this study targeting Middle Eastern adolescents in Malaysia have significant implications for public health in the country. By targeting adolescents, this program can help establish healthy habits early in life, potentially reducing the long-term burden of obesity related health issues. The focus on Middle Eastern adolescents in Malaysia underscores the importance of culturally sensitive health education. Tailoring interventions to specific ethnic groups can enhance their effectiveness by addressing cultural barriers and preferences. Overall, This educational intervention highlighted the potential for a targeted Healthy Lifestyle educational programme to improve public health outcomes in Malaysia by addressing specific health challenges among diverse populations.

There are several limitations to this study. First, data were collected using a self-report questionnaire. This method may introduce bias, as participants might provide socially desirable responses rather than candid answers. Therefore, the authenticity of the responses cannot be fully guaranteed. However, the presence of the researcher during questionnaire administration may have helped participants better understand the questions. Additionally, this study was conducted in four Arabic schools in Malaysia. To achieve better representation, future research should expand the sampling frame to include more Arabic schools across Malaysia and other regions. Further studies could also explore the knowledge, attitudes, and practices related to nutrition, physical activity, and healthy behaviours by involving a larger and more diverse sample of schools and adolescent students from different areas.

## Conclusion

The health educational intervention demonstrated significant positive impacts on knowledge, attitude, practice, and physical activity among Middle Eastern adolescents in Malaysia. There were notable differences in the mean scores for knowledge, attitude, practice, and physical activity levels between IG and CG across the three study time points, as identified by a GEE analysis. Given its potential to enhance knowledge, attitudes, practices, and physical activity throughout the intervention stages, this educational programme could serve as an effective strategy for promoting healthy behaviours among adolescents. The findings of this study provide a comprehensive overview of the Healthy Lifestyle intervention programme for Middle Eastern students, aiming to prevent or reduce obesity and promote healthier behaviours in the future. These results can serve as a foundation for developing future Healthy Lifestyle intervention programmes, offering valuable information that can be used as a reference or baseline data.

## Figures and Tables

**Table 1:** Descriptive statistics of the IG (*n* = 116) and CG (*n* = 119) at pre-intervention stage

Variables		IG	CG	*Z*/*χ*^2^	*P*-value

		Median [IQR] or *n* (%)			
Gender	Male	54.0 (46.6)	62.0 (52.1)	0.724^b^	0.395
	Female	62.0 (53.4)	57.0 (47.9)		

Grade	Seventh	51.0 (44.0)	58.0 (48.7)	0.538^b^	0.463
	Eighth	65.0 (56.0)	61.0 (51.3)		

Nationality	Yemeni	24.0 (20.7)	80.0 (67.2)	87.698^b^	< 0.001*
	Saudi	67.0 (57.8)	4.0 (3.4)		
	Others**	25.0 (21.6)	35.0 (29.4)		

Mother’s education	Intermediate or lower	9.0 (7.8)	17.0 (14.3)	4.306^b^	0.116
	High school	46.0 (39.7)	29.0 (24.4)		
	Undergraduate degree or higher	61.0 (52.6)	73.0 (61.3)		

Father’s education	Intermediate or lower	3.0 (2.6)	4.0 (3.4)	0.761^b^	0.684
	High school	19.0 (16.4)	15.0 (12.6)		
	Undergraduate degree or higher	94.0 (81.0)	100.0 (84.0)		

Household income^c^	< RM 5,000	26.0 (22.4)	54.0 (45.4)	24.683^b^	< 0.001*
	RM 5,000 to RM 14,999	47.0 (40.5)	51.0 (42.9)		
	≥RM 15,000	43.0 (37.1)	14.0 (11.8)		

Age		14.0 [1]	14.0 [1]	−0.728^a^	0.467

Number of siblings		3.0 [3]	3.0 [2]	−2.295^a^	0.022*

Household size		6.0 [2]	5.0 [1]	−1.492^a^	0.136

Knowledge score		11.5 [9]	10.0 [14]	−1.503^a^	0.133

Attitude score		67.0 [7]	66.0 [5]	−1.590^a^	0.112

Practice score		60.0 [10]	62.0 [8]	−0.606^a^	0.545

Physical Activity score		1.8 [0.8]	1.8 [0.8]	−0.542^a^	0.588

*n* = sample size; IG = intervention group; CG = control group; IQR= interquartile range;

a Mann-Whitney U test (*z*);

b Chi-square test (*χ*^2^);

* Significant result at *P* < 0.05;

c Malaysian ringgit = USD 4.70;

** Others including Iraqi, Palestinian, Syrian, Jordanian, Sudanese, Egyptian, Algerian, Moroccan, Tunisian, Libyan and Somalian

**Table 2:** Descriptive statistics of the mean score of knowledge, attitude, practice and physical activity of the IG and CG across time

Group	Variables	Pre-intervention (mean [SD])	Post-intervention (mean [SD])	Follow-up (mean [SD])
IG	Knowledge	11.37 (6.56)	24.82 (4.40)	24.41 (4.68)
	Attitude	69.15 (6.54)	84.59 (8.88)	84.77 (8.23)
	Practice	59.98 (9.58)	71.41 (6.23)	70.88 (6.51)
	Physical activity	1.90 (0.59)	2.25 (0.42)	2.23 (0.42)

CG	Knowledge	9.85 (7.11)	12.54 (5.15)	14.25 (3.74)
	Attitude	67.61 (8.22)	68.37 (8.38)	69.30 (9.39)
	Practice	58.65 (8.78)	61.50 (7.97)	62.68 (9.55)
	Physical activity	1.83 (0.51)	1.89 (0.50)	1.87 (0.49)

SD = standard deviation; IG = intervention group; CG = control group

**Table 3:** Generalised estimating equation analysis on the mean score of knowledge, attitude and practice

Variables	Source	Wald Chi-Square	*df*	*P*-value
Knowledge	Time point	433.834*	2	< 0.001
	Group	199.259*	1	< 0.001
	Time point * Group	225.302*	2	< 0.001

Attitude	Time point	320.420*	2	< 0.001
	Group	142.382*	1	< 0.001
	Time point * Group	240.788*	2	< 0.001

Practice	Time point	266.964*	2	< 0.001
	Group	51.142*	1	< 0.001
	Time point * Group	86.566*	2	<0.001

Physical activity	Time point	94.633*	2	< 0.001
	Group	19.415*	1	< 0.001
	Time point * Group	55.491*	2	< 0.001

*df* = degree of freedom; Time = Pre-intervention, post-intervention, three-month follow-up; Group = intervention group vs. control group; Time*Group = interaction time and group;

* Significant result at *P* < 0.05

**Table 4:** Pairwise comparison of the mean score of knowledge, attitude, practice and physical activity scores of the intervention (*n* = 111) and control (*n* = 112) groups over the study period

Variables	Group	(I) Test	(J) Test	Mean difference (I–J)	SE	*P*-value	95% CI for difference		*d*

							LB	UB	
Knowledge	IG	Pre-intervention	Post-intervention	−13.4483*	0.67455	< 0.001	−15.4282	−11.4683	2.75
		Pre-intervention	Follow-up	−13.0345*	0.70941	< 0.001	−15.1167	−10.9522	
		Post-intervention	Follow-up	0.4138	0.21154	0.757	−0.2071	1.0347	

	CG	Pre-intervention	Post-intervention	−2.6891*	0.39177	< 0.001	−3.8390	−1.5391	0.79
		Pre-intervention	Follow-up	−4.4034*	0.53746	< 0.001	−5.9809	−2.8258	
		Post-intervention	Follow-up	−1.7143*	0.33679	< 0.001	−2.7028	−0.7257	

Attitude	IG	Pre-intervention	Post-intervention	−15.4483*	0.80032	< 0.001	−17.7974	−13.0992	2.62
		Pre-intervention	Follow-up	−15.6207*	0.81040	< 0.001	−17.9994	−13.2420	
		Post-intervention	Follow-up	−0.1724	0.46763	1.000	−1.5450	1.2002	

	CG	Pre-intervention	Post-intervention	−0.7563	0.56044	1.000	−2.4013	0.8887	0.23
		Pre-intervention	Follow-up	−1.6891	0.68915	0.214	−3.7119	0.3337	
		Post-intervention	Follow-up	−0.9328	0.66538	1.000	−2.8858	1.0202	

Practice	IG	Pre-intervention	Post-intervention	−11.4310*	0.62739	< 0.001	−13.2725	−9.5895	1.94
		Pre-intervention	Follow-up	−10.8966*	0.65838	< 0.001	−12.8290	−8.9641	
		Post-intervention	Follow-up	0.5345	0.25702	0.563	−0.2199	1.2889	

	CG	Pre-intervention	Post-intervention	−2.8487*	0.68048	< 0.001	−4.8461	−0.8514	0.57
		Pre-intervention	Follow-up	−4.0336*	0.86395	< 0.001	−6.5695	−1.4978	
		Post-intervention	Follow-up	−1.1849	0.85508	1.000	−3.6947	1.3250	

Physical activity	IG	Pre-intervention	Post-intervention	−0.3546*	0.03910	< 0.001	−0.4693	−0.2398	0.23
		Pre-intervention	Follow-up	−0.3294*	0.03758	< 0.001	−0.4397	−0.2191	
		Post-intervention	Follow-up	0.0252*	0.00841	0.041	0.0005	0.0499	

	CG	Pre-intervention	Post-intervention	−0.0550	0.01938	0.069	−0.1118	0.0019	0.04
		Pre-intervention	Follow-up	−0.0370	0.01481	0.189	−0.0805	0.0065	
		Post-intervention	Follow-up	0.0180	0.02508	1.000	−0.0556	0.0916	

(I) Test = time; (J) Test = time; Mean difference (I–J) = difference between time; SE = standard error of the sample; CI = confidence interval is arranged of population values; LB = lower bound; UB = upper bound; *d* = Cohen effect size; IG = intervention group; CG = control group;

* The mean difference is a significant result at *P* < 0.05

**Table 5:** Pairwise comparison between the intervention (*n* = 111) and control (*n* = 112) groups for assessments of knowledge, attitude, practice and physical activity

Variables	Test	(I) Group	(J) Group	Mean difference (I–J)	SE	*P*-value	95% CI for difference		*d*

							LB	UB	
Knowledge	Pre-intervention	IG	CG	1.5220	0.88868	1.000	−1.0865	4.1304	0.22
	Post-intervention	IG	CG	12.2812*	0.62124	< 0.001	10.4577	14.1046	2.56
	Follow-up	IG	CG	10.1531*	0.55133	< 0.001	8.5348	11.7713	2.40

Attitude	Pre-intervention	IG	CG	1.5331	0.96359	1.000	−1.2952	4.3614	0.21
	Post-intervention	IG	CG	16.2251*	1.12186	< 0.001	12.9322	19.5180	1.88
	Follow-up	IG	CG	15.4647*	1.14581	< 0.001	12.1016	18.8279	1.75

Practice	Pre-intervention	IG	CG	1.3357	1.19446	1.000	−2.1703	4.8417	0.14
	Post-intervention	IG	CG	9.9180*	0.92787	< 0.001	7.1945	12.6415	1.41
	Follow-up	IG	CG	8.1986*	1.05982	< 0.001	5.0879	11.3094	1.00

Physical activity	Pre-intervention	IG	CG	0.0683	0.07211	1.000	−0.1434	0.2799	0.13
	Post-intervention	IG	CG	0.3679*	0.05974	< 0.001	0.1926	0.5432	0.78
	Follow-up	IG	CG	0.3607*	0.05956	< 0.001	0.1859	0.5355	0.79

Mean difference (I–J) = difference between groups; SE = standard error of the sample; CI = confidence interval is arranged of population values; LB = lower bound; UB = upper bound; *d* = Cohen effect size; IG = intervention group; CG = control group;

* The mean difference is a significant result at *P* < 0.05
